# Dynamic Change of Global and Local Information Processing in Propofol-Induced Loss and Recovery of Consciousness

**DOI:** 10.1371/journal.pcbi.1003271

**Published:** 2013-10-17

**Authors:** Martin M. Monti, Evan S. Lutkenhoff, Mikail Rubinov, Pierre Boveroux, Audrey Vanhaudenhuyse, Olivia Gosseries, Marie-Aurélie Bruno, Quentin Noirhomme, Mélanie Boly, Steven Laureys

**Affiliations:** 1 Department of Psychology, University of California Los Angeles, Los Angeles, California, United States of America; 2 Brain Injury Research Center (BIRC), Department of Neurosurgery, David Geffen School of Medicine at UCLA, Los Angeles, California, United States of America; 3 Brain Mapping Unit, Department of Psychiatry, University of Cambridge, Cambridge, United Kingdom; 4 Churchill College, University of Cambridge, Cambridge, United Kingdom; 5 Coma Science Group, Cyclotron Research Center, University of Liège, Liège, Belgium; Brain and Spine Institute (ICM), France

## Abstract

Whether unique to humans or not, consciousness is a central aspect of our experience of the world. The neural fingerprint of this experience, however, remains one of the least understood aspects of the human brain. In this paper we employ graph-theoretic measures and support vector machine classification to assess, in 12 healthy volunteers, the dynamic reconfiguration of functional connectivity during wakefulness, propofol-induced sedation and loss of consciousness, and the recovery of wakefulness. Our main findings, based on resting-state fMRI, are three-fold. First, we find that propofol-induced anesthesia does not bear differently on long-range versus short-range connections. Second, our multi-stage design dissociated an initial phase of thalamo-cortical and cortico-cortical hyperconnectivity, present during sedation, from a phase of cortico-cortical hypoconnectivity, apparent during loss of consciousness. Finally, we show that while clustering is increased during loss of consciousness, as recently suggested, it also remains significantly elevated during wakefulness recovery. Conversely, the characteristic path length of brain networks (i.e., the average functional distance between any two regions of the brain) appears significantly increased only during loss of consciousness, marking a decrease of global information-processing efficiency uniquely associated with unconsciousness. These findings suggest that propofol-induced loss of consciousness is mainly tied to cortico-cortical and not thalamo-cortical mechanisms, and that decreased efficiency of information flow is the main feature differentiating the conscious from the unconscious brain.

## Author Summary

One of the most elusive aspects of the human brain is the neural fingerprint of the subjective feeling of consciousness. While a growing body of experimental evidence is starting to address this issue, to date we are still hard pressed to answer even basic questions concerning the nature of consciousness in humans as well as other species. In the present study we follow a recent theoretical construct according to which the crucial factor underlying consciousness is the modality with which information is exchanged across different parts of the brain. In particular, we represent the brain as a network of regions exchanging information (as is typically done in a comparatively young branch of mathematics referred to as graph theory), and assess how different levels of consciousness induced by anesthetic agent affect the quality of information exchange across regions of the network. Overall, our findings show that what makes the state of propofol-induced loss of consciousness different from all other conditions (namely, wakefulness, light sedation, and consciousness recovery) is the fact that all regions of the brain appear to be functionally further apart, reducing the efficiency with which information can be exchanged across different parts of the network.

## Introduction

Despite the centrality of consciousness to our experience, no agreement has yet emerged on which aspects of brain function underlie its presence, and what changes are connected to its disappearance in the healthy brain (e.g., during sleep) as well as in pathological conditions (e.g., coma). As a consequence, we are currently hard pressed to answer even basic questions concerning the presence, absence, degree and nature of the phenomenon of consciousness in humans and other species [1]. As experimental investigations into this domain have increased, a number of proposals have been put forth to characterize the neural fingerprint of consciousness. According to some views, the crucial feature underlying consciousness is the presence of specific patterns of activations, such as the presence of competing assembly of cells, or ‘neural coalitions’ [2], synchronization of neural activity in specific frequency bands [3], [4], or the level of spontaneous oscillatory activity, at fast frequencies, in the thalamo-cortical system [5]. According to other proposals, consciousness is related to the spread and reverberation of information across the neural system, and in particular within specific regions in parietal and frontal cortices [6], [7] – although the scope of this view is mostly relevant to the idea of conscious availability of content to a neural system, as compared to the more general “state of consciousness” of a neural system [8]. Finally, a recently proposed view [1], [9], stresses the importance of evaluating not the degree of correlation among different (often long-range) regions, but rather the degree of information present and the extent to which information is integrated across the nodes of a system.

In the present work we look at spontaneous low-frequency fluctuations in the functional magnetic resonance imaging (fMRI) signal [10], [11], to assess the relationship between different states of consciousness and basic principles of information processing (as captured by the blood oxygenation level dependent signal; i.e., BOLD). The analysis of spontaneous fluctuations of the BOLD signal has been fruitfully employed to explore consciousness-related changes in clusters of temporally coherent regions during sedation [12], [13], sleep [14]–[16], and in the pathological brain [17], [18]. In particular, associations within specific networks of regions have been found to be monotonically modulated by consciousness [19]–[21], consistent with some theoretical views [3]–[5]. This idea, however, clashes with reports of increased cross-regional correlation concurrent with decrease or loss of consciousness [22], [23], suggesting the importance of characterizing not just the strength but also the quality of information processing within a system [1], [24].

Following this idea, we employ previously collected resting-state fMRI data [21] to assess, in 12 healthy volunteers, the dynamic change of governing principles of brain organization during wakefulness (W), propofol-induced sedation (S) and loss of consciousness (LOC), as well as after consciousness recovery (R), a dynamic approach that has been recently advocated for [25]. In particular, we focus on the change of global and local topological metrics of information processing across conditions [26]–[28], a technique that has been successfully employed to characterize and model dynamics within physical [29], biological [30] and social systems [31], and that has been shown to capture specific aspects of brain organization in the maturing, healthy adult, and pathological brain [32]–[36]. A particularly appealing aspect of this technique in the context of studies of consciousness is the parallel between the measures it offers, focused on characterizing how information is exchanged and propagated through a network, and theories of consciousness that stress the centrality of how information is treated and integrated within the brain [1], [9].

As detailed below, we report three main findings. First, contrary to a recent report [25], we find that long- and short-range connections are not differentially affected by sedation. Second, employing a support vector machine (SVM) classifier, we dissociate the thalamo-cortical and cortico-cortical hyperconnectivity observed during sedation from the cortico-cortical hypoconnectivity observed during loss of consciousness. Finally, contrary to results in other species [37], we find significant global changes in the (functional) topological organization of the brain during sedation. However, we show that normalized clustering, the global metric that was previously reported to be sensitive to the loss of consciousness [25], remains significantly elevated also through post-sedation recovery of wakefulness. Conversely, we find that a strong decrease in efficiency of information distribution (defined as the inverse of the characteristic path length – see Materials and Methods) is the only unambiguous marker of propofol-induced loss of consciousness.

## Results

### Network Description

**Figure 1:**
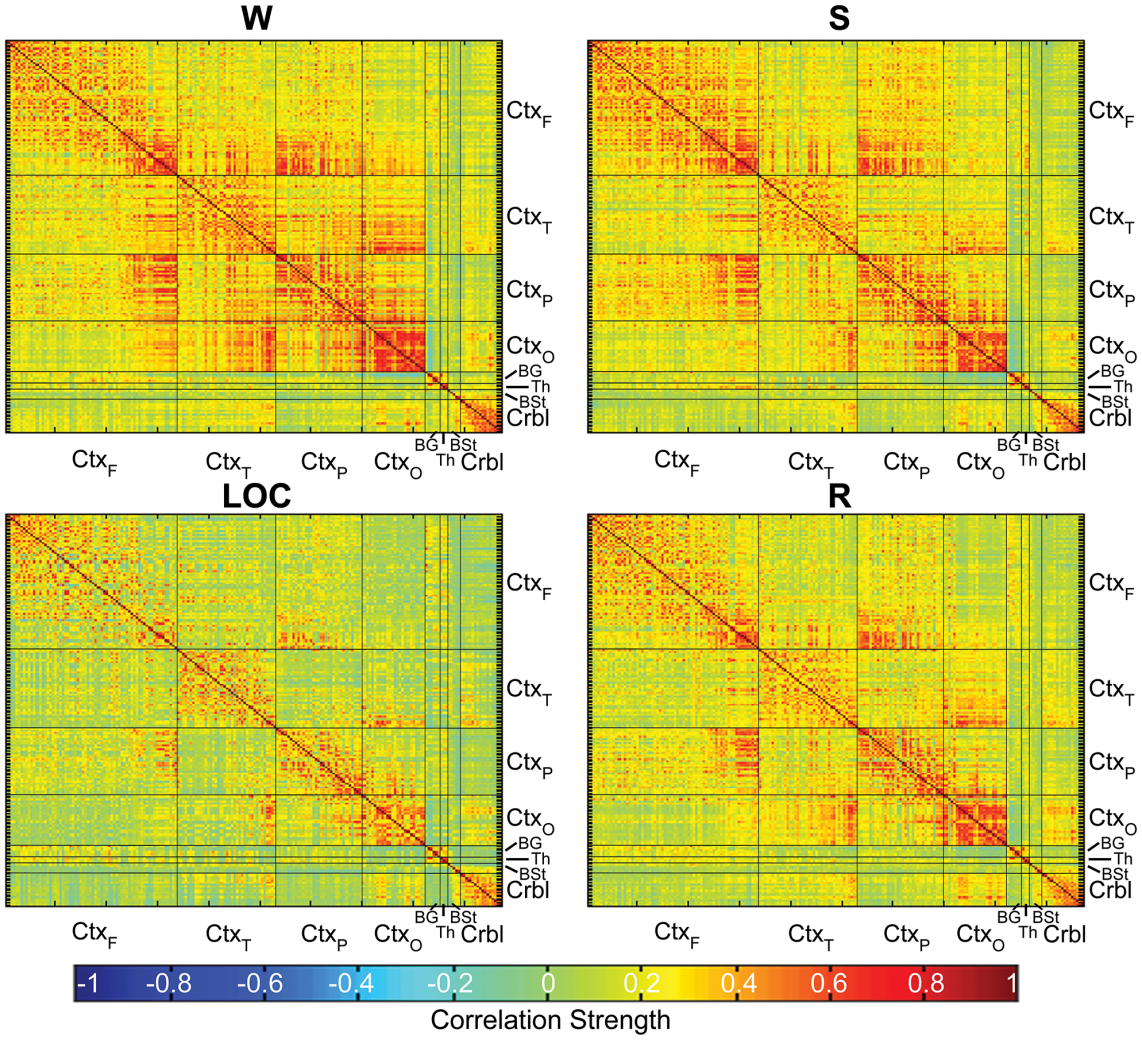
Mean connectivity matrices for each condition. Within each subdivision/lobe, ROIs appear in a rostral-to-caudal fashion. Abbreviations: 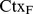: cortex, frontal lobe; 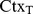: cortex, temporal lobe; 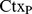: cortex, parietal lobe; 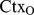: cortex, occipital lobe; BG: basal ganglia; Thl: thalamus; BS: brainstem; Crbl: cerebellum.

**Figure 2:**
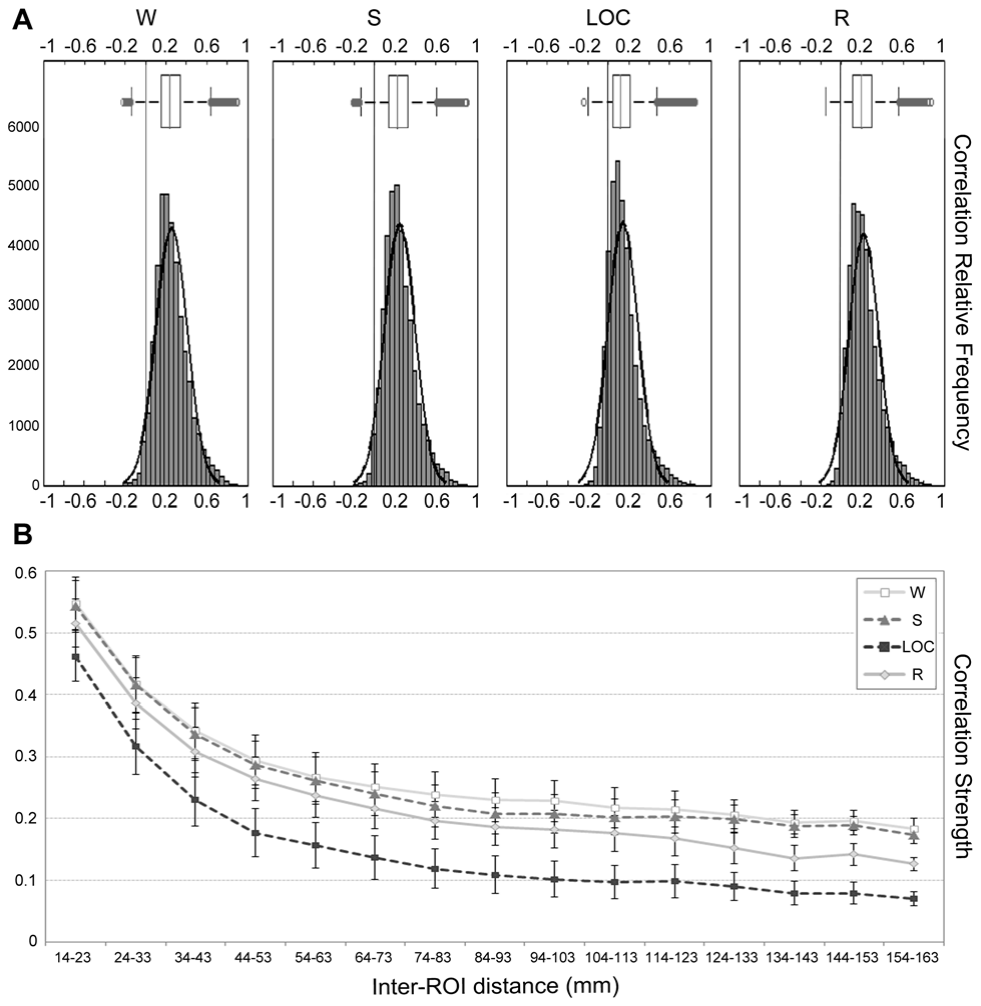
Correlations description. (a) Frequency distribution of ROI correlations for each condition (and boxplot); (b) Strength of ROI correlations for each condition as a function of inter-ROI distance (for display purposes regions are binned in fifteen-9 mm distance groups).

The average connectivity matrices and the frequency distribution of (average) correlations for each condition are shown in Figure 1 and Figure 2a, respectively. According to a two-sample Kolmogorov-Smirnov goodness-of-fit test, the distribution of positive and negative correlations are significantly different for all pairwise comparisons (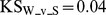; 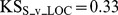; 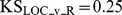; 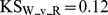; all 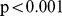). In all four conditions about 80% of correlations were between 0 and 0.4. LOC, however, exhibited a leftwards shift of the distribution, as shown by the median correlation value of 0.11, as compared to 0.23, 0.22, and 0.19 for W, S, and R, respectively. Furthermore, 14% of correlations in the LOC condition were negative, as compared to about 2% in all other conditions, while only 6% were above 0.4, versus 17%, 14% and 11% for W, S, and R, respectively. To assess whether correlations between areas at different distances were unequally affected by the level of consciousness, we employed a repeated measures ANCOVA with one within-subjects variable (i.e., condition) with four levels (W, S, LOC, R), and inter-ROI distance as a covariate (with distance defined as the 3-dimensional Euclidean distance between the baricenter of each ROI; see Figure 2b) to predict correlation strength. As expected, we found a significant effect of condition (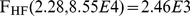, 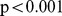), indicating that correlation strength systematically varied across conditions. Specifically, W consistently exhibited the strongest average correlation level, across all bins, followed by S and R, while LOC consistently exhibited the weakest average correlation across all bins. We also found a significant effect of distance (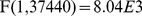, 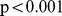), indicating that, as shown in Figure 2b, the average correlation strength decreased with distance. In addition to the two main effects, we also found a significant interaction between condition and distance (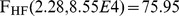, 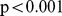), indicating an uneven effect of condition on links of different length. However, when we followed up this significant interaction with a set of separate repeated measures ANOVAs (one per each bin) we found that it was entirely driven by the absence of a significant difference between W and S for the first 3 bins (out of 15; i.e., regions closer than 3.4 cm). With this exception, the effect of propofol was remarkably consistent at all other connection lengths (particularly with respect to the crucial condition – i.e., loss of consciousness – where no difference was found across connection length). Indeed, at all other bins the four conditions were found to be significantly different from each other, based on estimated marginal means and a Sidak correction for multiple comparisons. The observation of a small effect of distance on connection strength across levels` of sedation is also consistent with the extremely low effect size observed for the interaction between condition and distance in the overall ANOVA (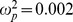), and strengthens the idea that, overall, connection size had a minimal effect on correlation strength – something that is immediately clear from Figure 2b.

### Network Classification

**Figure 3:**
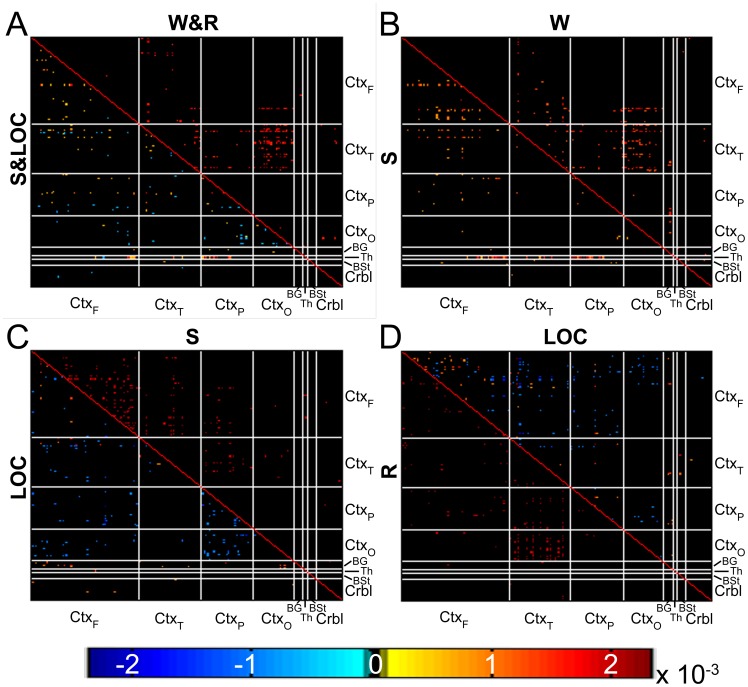
Classification results. Top 1% connections contributing to the group SVM classification of (A) all wakefulness conditions (W&R) vs. all sedation conditions (S&LOC); (B) W vs. S; (C) S vs. LOC; (D) LOC vs. R. For each comparison, the upper triangle shows the connections contributing to correctly classifying the first condition, the lower triangle shows the connections contributing to correctly classifying the second condition. Red connections indicate positive correlations contributing to the correct classification of a condition, blue connections indicate negative correlations contributing to the correct classification of a condition. (Since classifications are relative to a comparison group, top classifying nodes for a given condition may differ according to what group it is classified against.) See Figure 1 for abbreviations.

Results for the classification of brain networks (i.e., correlation matrices) are reported in Table 1 and Figure 3. At a global level, the SVM algorithm classified successfully states of wakefulness (W & R) versus states of sedation (S & LOC) with high accuracy, sensitivity and specificity (all above 83.33%; 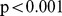). The same level of classification was also achieved when comparing contiguous brain states (namely, W vs. S; S vs. LOC; and LOC vs. R; see Table 1 for a detailed report of accuracy, specificity, sensitivity and significance for each). Conversely, wakefulness (W) and wakefulness recovery (R) could not be successfully distinguished from each other (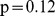). (For completeness the two remaining classifications, namely W vs. LOC and S vs. R, are reported in Figure S1.)

**Table 1:** Results of brain network group classification with SVM algorithm.

Comparison	N (per group)	Accuracy (%)	Sensitivity (%)	Specificity (%)	p-value
(W&R) vs. (S&LOC)	24	85.42	87.50	83.33	p<0.001*
W vs. S	12	83.33	83.33	83.33	p<0.001*
S vs. LOC	12	91.67	100.00	83.33	p<0.001*
LOC vs. R	12	87.50	83.33	91.67	p<0.001*
W vs. R	12	62.50	58.33	66.67	p = 0.120

‘*’ indicates the classification survives Bonferroni correction.

**Figure 4:**
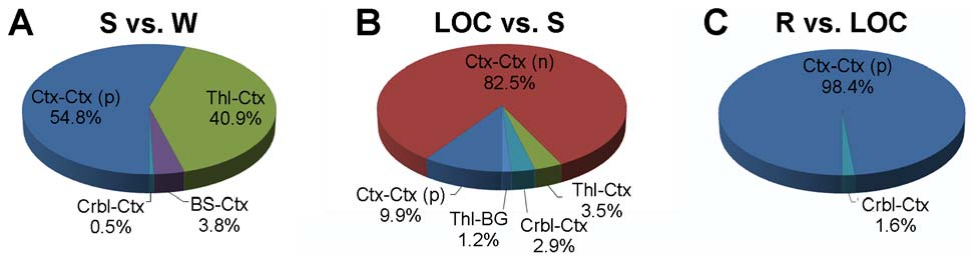
Distribution of classifying connections. Distribution of the top 1% connections contributing to correct SVM classification for (A) S vs. W, (B) LOC vs. S (middle), and (C) R vs. LOC. See Figure 1 for abbreviations; in addition ‘p’ indicates positive correlations; ‘n’ indicates negative correlations.

At the local level, accurate classification of each transition relied on different sets of edges within each brain graph (see Tables S1 and S2 for full details). In particular, as depicted in Figure 3b, and more in detail in Figure 4a, the edges mostly contributing to correctly classifying S versus W included positive cortico-cortical (54.8%) and thalamo-cortical (40.9%) connections, as well as a minority of cerebello-cortical (0.5%) and striato-cortical (3.8%) connections. Conversely, as depicted in Figures 3c and 4b, the distribution of connections correctly classifying LOC, as compared to S, mostly included negative cortico-cortical connections (82.5%), as well as a minority of positive cortico-cortical (9.9%), thalamo-cortical (3.5%), cerebello-cortical (2.9%) and thalamo-striatal (1.2%) connections. Notably, when tested statistically, the allocation of classifying edges for these two transitions are significantly different (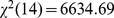, 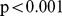). Finally, as shown in Figures 3d and 4c, as compared to LOC, classification of R was almost entirely based on the re-emergence of positive cortico-cortical connections (98.4%) as well as a small minority of cerebello-cortical connections (1.6%).

### Network Analysis

#### Global metrics

**Figure 5:**
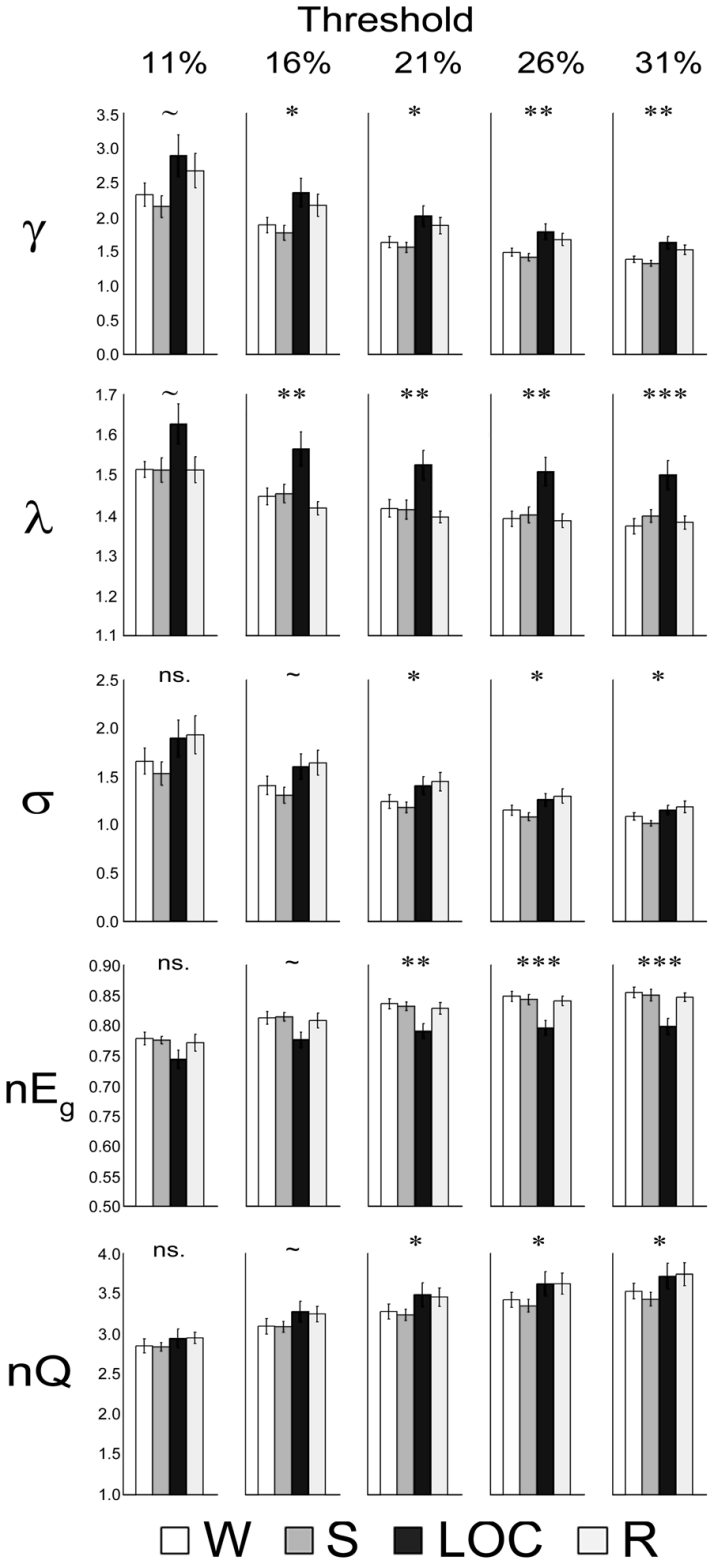
Global metrics. Average normalized global network metrics for each condition at each threshold (bars depict standard error). Abbreviations: Clustering, 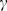; characteristic path length, 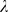; small-worldness, 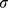; efficiency, 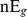; modularity, nQ. Significance level: 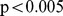 ‘***’; 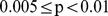 ‘**’; 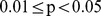 ‘*’; 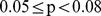 ‘∼’; 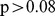 ‘ns’.

Normalized global network metrics for each condition, across all thresholds, are reported in Figure 5. (As described in the Materials and Methods section, all the following network properties are computed on weighted matrices using weight-conserving algorithms. This approach, which is a departure from previous research in this field [25], [37], is motivated by the fact that binary matrices equally assigning a value of ‘1’ to all suprathreshold edges regardless of their connection strength are susceptible to false short paths which may significantly affect results [38] – see the Materials and Methods section for further discussion. In the Results and Discussion sections the ‘
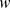
’ superscript is omitted for notation simplicity.)

Overall, the global repeated measures ANOVA indicated a significant effect of condition on normalized clustering (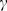; 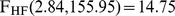, 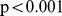, 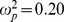). The threshold factor was also significant (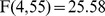, 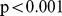, 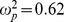), as expected, but did not interact with the condition factor (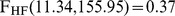, 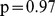), indicating that the effect of the level of sedation on this measure is robust to the thresholding procedure. Follow-up pairwise comparison indicated that the effect of condition was mainly due to W and S exhibiting significantly less clustering than LOC and R, while no significant difference was found between either the first two or the latter two conditions. The follow-up 1-way ANOVAs (one per threshold) replicated the overall results at all thresholds, although it was only marginally significant at the lowest density threshold (i.e., 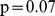; see Figure 5, top row).

The level of sedation also affected the normalized characteristic path length (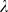; 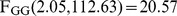, 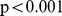, 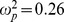). Threshold level was also significant (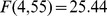, 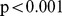, 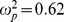), but did not show any interaction with the level of sedation (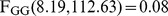). Pairwise comparisons indicated that the only significant difference occurred between LOC and all other conditions, with the state of unconsciousness exhibiting significantly greater (normalized) characteristic path length. These results were also replicated, at each threshold, with the 1-way ANOVAs (although the effect was only marginally significant at the lowest density threshold; i.e., 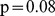; see Figure 5, second row).

The effect of condition on small-world properties (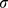) generally mirrored that seen for 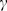, with a significant effect of condition (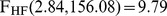, 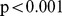, 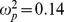), a significant effect of threshold (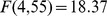, 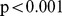, 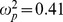) and no significant interaction between the two factors (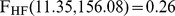, 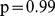). Follow-up pairwise comparisons indicated that, as for 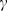, the first two conditions (i.e., W and S) were not significantly different from each other, but both exhibited significantly smaller 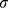 than the last two conditions (LOC and R), which were not significantly different from each other. In the 1-way ANOVAs, the same pattern was numerically detected at each threshold, but it was only significant at the three highest density thresholds (i.e., 21%, 26% and 31%), non-significant at the lowest density threshold (i.e., 11%) and marginally significant at the second lowest threshold (i.e., 16%; see Figure 5, third row).

Consistent with the results for 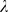, a significant effect of condition was also found on normalized global efficiency (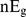; 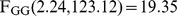, 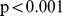, 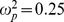). Threshold also exhibited the expected main effect (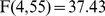, 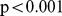, 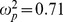), but again no interaction was observed with the level of sedation (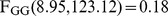, 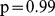). Pairwise comparisons indicated that in LOC efficiency is significantly decreased, as compared to all other conditions. The general pattern was replicated at each threshold individually, but it was only significant for the three highest density thresholds (i.e., 21%, 26% and 31%), marginally significant at the second lowest density (i.e., 16%; p = 0.06) and non-significant at the lowest density threshold (i.e., 11%; see Figure 5, fourth row).

Finally, normalized mean modularity (nQ) was also significantly affected by condition (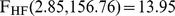, 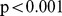, 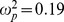) and threshold (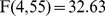, 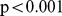, 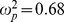), with no significant interaction between the two factors (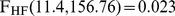, 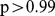). Pairwise comparison indicated that the effect of condition was mainly due to W and S exhibiting significantly less modularity than LOC and R, while no significant difference was found between either the first two or the latter two conditions. This same effect was seen in the follow-up ANOVAs at the three highest density thresholds (i.e., 21%, 26% and 31%), while only a marginal effect was seen at the second threshold (i.e., 16%) and no effect at the lowest threshold (i.e., 11%). With respect to the mean number of modules uncovered in each condition, at the global level we find a marginally significant effect of sedation (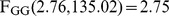, 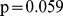), although it exhibited a very small effect size (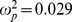). Pairwise comparison indicated that the only difference was observed between S and LOC, with the latter showing a lower number of modules than the former. Consistent with the effect size statistic, however, although the same numerical trend was observed at each individual threshold (see in Table 2), it was never found to be significant in any of the follow-up 1-way ANOVAs.

**Table 2:** Mean number of modules for each condition at each density threshold (SD reported in parenthesis).

threshold	W	S	LOC	R
11%	15.00 (9.33)	14.72 (5.90)	10.60 (5.15)	11.26 (6.99)
16%	8.81 (3.72)	9.39 (2.94)	7.03 (2.79)	7.92 (4.96)
21%	6.81 (2.46)	6.95 (2.06)	5.30 (1.74)	6.42 (3.73)
26%	5.34 (1.46)	5.55 (1.59)	4.95 (1.44)	5.09 (2.38)
31%	4.71 (1.28)	4.85 (1.00)	4.56 (0.99)	4.54 (1.57)

#### Local metrics

**Figure 6:**
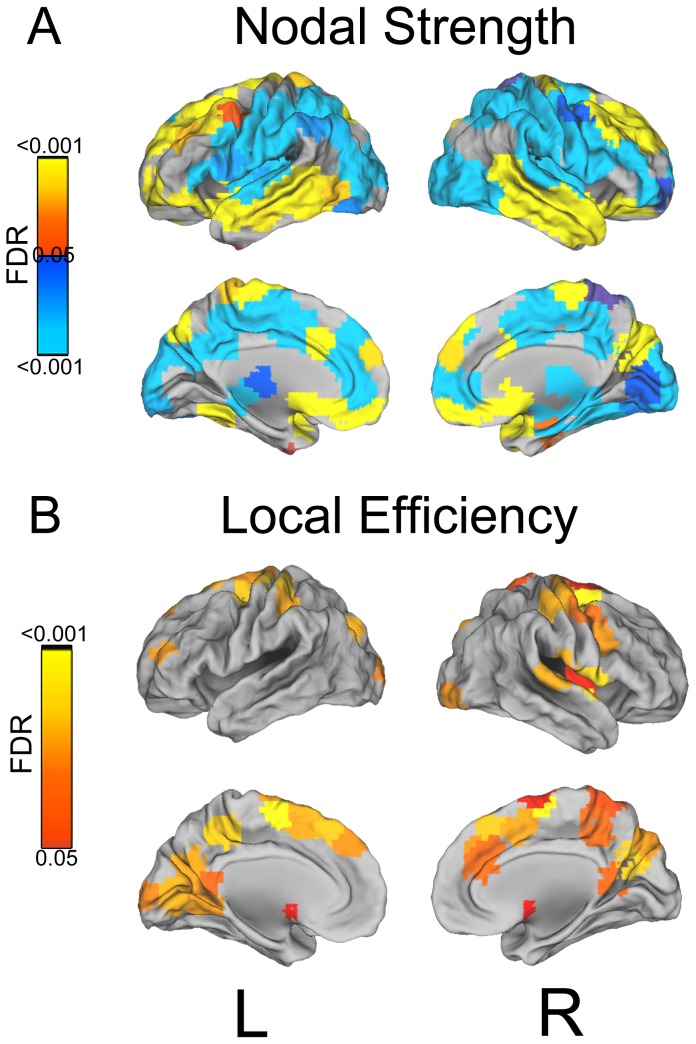
Local metrics. Regions displaying a significant effect of condition on local metrics. (a) Nodal strength (yellow-red colors indicate regions in which degree was stronger, on average, for the S and LOC conditions, while blue-lightblue colors indicate regions in which degree was stronger for W and R). (b) Local efficiency (yellow-red colors indicate regions in which the measure is stronger, on average for the LOC and R conditions). Color intensity is assigned on the basis of the (FDR adjusted) p-value for the condition factor in the 2-way repeated measures ANOVA. (Surface rendering was performed using Caret [98].)

With respect to local metrics, results for nodal strength and local efficiency are depicted in Figure 6. The level of sedation had a significant effect on nodal strength across a wide variety of lateral and midline regions. Overall, two main patterns were detected. In some regions (shown in blue in Figure 6a), local strength was stronger (across thresholds and subjects) during W and R. This U-shape pattern was detected across a wide number of regions throughout the midline as well as in occipital, parietal and latero-ventral prefrontal-cortices (consistent with previous results [25]). In other regions (shown in yellow-red in Figure 6a), local strength was stronger during S and LOC, as compared to the other two conditions. This inverted U-shape pattern was mainly detected in temporal cortex, ventro-medial prefrontal cortex, and the apical aspect of prefrontal cortex. The effect of sedation on local efficiency (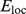) was, as expected (cf. [39]), consistent with the clustering results. All regions in which a significant effect of condition was detected exhibited greater efficiency (on average, across subjects and density thresholds) in LOC and R. As shown in Figure 6b, condition mainly affected the local efficiency of regions within the medial section of parietal and frontal cortices.

## Discussion

In this study we assessed propofol-induced changes in patterns of connectivity, as well as in global and local governing principles of brain organization, during wakefulness, sedation, loss of consciousness, and wakefulness recovery. Our results contribute to a growing literature addressing the topological organization of the human brain [26], [38], the changes in functional architecture accompanying the loss of consciousness [16], [25], [37], as well as a specific hypothesis concerning the role of different subsystems in loss of consciousness [40], [41].

Overall, our main findings are three-fold. First, despite the frequently voiced idea that long-range connections play a key role in anesthesia-induced unconsciousness [40], we fail to find a substantial asymmetric decrease in cross-region correlation as a function of inter-regional distance. Average connectivity strength decreased monotonically with distance in approximately the same manner across conditions (with the sole exception of extremely short connections, below 34 mm, but only during the initial phase of sedation, and not during loss of consciousness). This finding runs counter to a recent report demonstrating an uneven effect of propofol-induced unconsciousness on short-range (i.e., 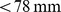) versus long-range (i.e., 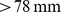) connections [25]. The only effect we detected concerned much shorter connections (i.e., 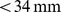), and was only found for the initial period of sedation, and not for the period of loss of consciousness. Whether the different result is to be attributed to methodological asymmetries (e.g., 2-timepoint versus 4-timepoint paradigms, the binning procedure, the use of different ROIs parcellation schemes) or to un-modelled third factors remains to be determined.

The second central aspect of our results directly addresses the discussion concerning the role of thalamo-cortical versus cortico-cortical circuits in propofol-induced unconsciousness [40], [41]. In particular, our SVM classification isolated increased thalamo-cortical and cortico-cortical synchronization as being maximally informative in the wakefulness versus sedation classification, suggesting a prominent role of this circuit in the initial stages of sedation, before the onset of unconsciousness. Conversely, correct classification of the state of loss of consciousness, as compared to sedation, overwhelmingly relied on negative cortico-cortical correlations. These findings support the view that propofol-induced loss of consciousness is more closely linked to cortico-cortical mechanisms rather than thalamo-cortical ones, as also suggested in a recent EEG effective connectivity study [41]. It is important to point out that our SVM classification is entirely based on the full matrix of ROI-to-ROI correlations and is, therefore, entirely data driven and blind to the existence of particular neural circuits or opposing hypothesis concerning their role in propofol-induced loss of consciousness. The observed major role of negative cortico-cortical connectivity in propofol-induced unconsciousness should be differentiated, however, from studies on pathological loss of consciousness in severe brain injury where post-mortem [42] and in-vivo [43] evidence highlights the role of thalamus in loss and recovery of consciousness [44], [45]. While further studies will have to directly address the issue, our findings are consistent with the suggestion that thalamus may be a necessary but not sufficient component in maintaining consciousness [41] consistent with the view that thalamic lesions might induce unconsciousness after severe brain injury by virtue of disconnecting an otherwise functioning cortex [46], [47].

The third result of our study concerns changes in governing principles of information processing during loss and recovery of consciousness. Contrary to a recent study in other species [37], we do find significant changes in global topological measures across levels of consciousness. Consistent with a previous report [25], we find that loss of consciousness is marked by an increase in normalized clustering (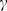), which measures the ‘cliquishness’ of brain regions, potentially indicating an increase in localized processing and thus a decrease of information integration across the brain. Our multi-stage design, however reveals that clustering remains significantly elevated (as compared to initial wakefulness and sedation) during post-anesthesia wakefulness recovery. This result shows that while it is true that clustering increases once consciousness is lost, it is not a sufficient marker of consciousness, something that the two-point design (i.e., initial wakefulness versus loss of consciousness) in Schröter *et al.* [25] could not reveal. On the other hand, we find that the normalized characteristic path length (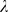) is significantly increased only during loss of consciousness, suggesting that during unconsciousness the efficiency of information distribution within the network is reduced (a finding that is consistent with a very recent study on loss of consciousness in sleep [16]). Whether this state of increased “functional distance” between regions is causal or consequent to propofol-induced loss of consciousness will have to be addressed in future research. As previously reported, the small-world architecture of brain networks (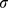) persisted (and in fact increased) in loss of consciousness [25], confirming the robustness of this core principle of organization of biological networks despite profound state changes [32]. Mirroring 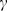, however, small-world architecture also remained significantly elevated during wakefulness recovery. Although much weaker, a similar effect of condition was also uncovered for normalized modularity (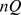). Finally, we remark that the presence of different results observed in the two propofol conditions (sedation and loss of consciousness) and, importantly, consciousness recovery, is consistent with the view that changes in global brain topology observed here and elsewhere [25], [37] are not simply due to drug exposure, but rather reflect brain state changes relating to the loss of consciousness, supporting a previously expressed view [25].

Beyond the global reorganization of brain topology, we also observed changes in local network topology. With respect to nodal strength, selected frontal and parietal regions along the midline, as well some lateral and opercular ROIs, appeared to be modulated by changes in the level of consciousness. In particular, regions in medial frontal and parietal cortices, along with occipital and lateral parietal, exhibited less nodal strength during sedation and loss of consciousness. Other regions, on the other hand, in temporal cortex especially, but also in dorsal and ventro-medial prefrontal cortex, exhibited the reverse pattern. Mirroring the result for 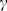, local efficiency appeared to be modulated mostly across midline parietal and prefrontal regions. Overall, this pattern of reorganization of local network topology is consistent with the view that propofol affects specific hubs central to normal/wakeful connectivity [48] which are also known to play a critical role in consciousness [49]–[51] and self-consciousness [52].

Taken together, our findings support the idea that (propofol-induced) loss of consciousness correlates with a change in the quality of information processing, and not only a change in the strength of connectivity across regions [1], [24]. In particular, dynamic reconfiguration of thalamo-cortical and cortico-cortical connections, and contemporaneous decrease of efficiency and increased local processing might affect the degree by which information can be effectively integrated across the brain [9].

In terms of theories of consciousness, these findings can be interpreted as making two contributions. First, the significant increase of cortico-cortical decorrelations during loss of consciousness is coherent with views of consciousness stressing the role of coherent reverberation and spread of neural activity [6], [7], particularly within fronto-parietal regions [5]. (We point out that, as shown in Tables S1 and S2, all fronto-parietal connections driving the correct classification of loss of consciousness, compared to sedation, are negative.) Second, our graph theoretic analysis further indicates that, in terms of network information processing, propofol-induced loss of consciousness is marked by a specific change in the quality of information exchange (i.e., decreased efficiency), consistent with the view that the specific modality with which information is exchanged within brain networks is crucial to the maintenance of a state of consciousness [1], [9].

Finally, it is important to stress that many of the methodological limitations expressed elsewhere concerning the interpretation of the blood oxygenation level dependent signal, as well as the current challenges tied to applying graph theory to brain measures previously discussed [25], [28], [32], [34], [37], also apply to our study. In particular, with respect to the implementation of graph-theory measures in neuroscience, several issues are still in search of resolution. Here, we believe it is important to stress five methodological considerations. First, as we note in the Materials and Methods section, most topological measures require thresholding of adjacency matrices, a procedure that presently lacks a defined standard approach (e.g., how many and which thresholds to employ) and might have important effects on the derived metrics [53]. While the real resolution of the issue will likely include measures that can be applied to fully connected matrices [28], we stress that our results were robust to the choice of threshold. Second, in contrast to some previous studies [25], we made use of weighted measures, a difference that might explain the divergence of results. For instance, we note that the observed between-group differences in our study were most pronounced at the lowest density thresholds (corresponding to least sparse networks), in contrast to many binary brain-network studies, in which between-group differences are most pronounced at the highest density thresholds (corresponding to most sparse networks) [54]. Many binary-network studies discard as many as 90–95% of all possible connections to elucidate the observed between-group differences [53] and it is likely that these more radical thresholding approaches are associated with substantial loss of connectivity information [55]. High thresholds are needed in binary studies because when weak and strong links surviving thresholding are equally assigned a value of 1, measures based on path length become susceptible to the creation of spurious long-distance short-cuts, which might obscure the architecture of strong connections and, thereby, important across-group differences [38]. It is therefore possible that the use of binary matrices in previous studies might have obscured the differences in characteristic path length that we have observed. Consistent with our findings, a recent study in the domain of sleep also uncovered loss of efficiency during unconsciousness [16]. Third, as discussed in the Materials and Methods section, because of the known effects of motion on graph theoretic analysis [56], [57], our sample was reduced to 12 volunteers. Although this sample size is within the boundaries of previous work on this same topic (e.g., N = 11 in [25], N = 20 in [37]) it does fall at the low end of the spectrum. Therefore, even though our analyses leverage on a statistically more powerful 4-point repeated measures design (as compared to the more typical two groups across-subjects comparison and two-points within subject design), future studies will have to confirm their generality. Nonetheless, we do stress that the effect-size analysis, which is robust to small samples, shows that our effects are of large magnitude, and that our results are consistent with previous reports [16]. Fourth, while we adopt the presently accepted mainstream interpretation of characteristic path length and global efficiency as measures of functional integration, we acknowledge that these interpretations have not been directly validated and are less trivial to make in networks where edges represent correlations and hence do not necessarily represent causal interactions or information flow [28]. Finally, it is important to stress that a recognized source of variance across results is the choice of ROIs [58], [59]. In particular, we employed more ROIs than in similar previous studies [25], [37], hence it is possible that some of the reported differences are due to the less granular parcellation schemes previously employed. Similarly, it is also possible that, if we had used an even greater number of ROIs, or based our networks on a voxel-wise analysis, results would have differed. However, it has been shown that simple binary decisions concerning the presence of certain network organizational parameters (e.g., small-worldness) are robust across different parcellation granularity [58]–[60]. Consistent with this finding, a recent study evaluating network properties during sleep reported a loss of efficiency during loss of consciousness that paralleles our own findings, despite the fact that their networks featured more than 3,700 nodes [16]. It should be stressed, however, that high granularity parcellations might yield quantitatively very different estimates of network properties, as compared to low granularity parcellations, and might allow topological features to be displayed more prominently [58], [59]. There is, however, an important conceptual difference that separates region-based networks from voxel-based networks [34], [61]. In our report, as in all region-based analyses of brain connectivity, network locality is conceived at a specific scale, determined by the coarseness of the employed parcellation. Hence, when we investigate local network properties, we are investigating topological features calculated over proximal brain regions. Conversely, voxel-wise networks assess locality within regions of the brain, an approach which has the potential advantage of capturing differences across regions of the brain in within- and between-connectivity [34], [61]. In this sense, region-based network analyses might be biased towards highlighting the properties of regions with widely distributed connections at a coarse scale, predominant in heteromodal association areas [62], and blind to local hierarchical connections more predominant in sensory cortical areas [63]. Voxel-based network analysis, instead, allow for examining inter-regional as well as intra-regional connectivity [34]. Nonetheless voxelwise parcellations might however pose conceptual difficulties with respect to computing global network properties because grid-like subdivisions do not generally respect boundaries or sizes of heterogeneous functional areas, an approach that might lead to mischaracterization of brain network function [64]. In conclusion, in interpreting our results (as any region-based network analysis with comparably sized, or larger, ROIs) it is thus important to keep in mind that our statements concerning changes in local topological features are intended as network-local, and do not necessarily reflect local changes at the brain physical level.

In sum, our findings show that changes in the level of consciousness induced by propofol affect basic organization principles and dynamics of information processing across the whole brain as well as within specific regions known to be involved in consciousness. In particular, we find that propofol-induced loss of consciousness is mostly associated with cortico-cortical mechanisms, as opposed to thalamo-cortical ones, and with a substantial decrease in the efficiency of information flow within the network. Future research will have to assess whether different anesthetic agents and pathology (e.g., brain trauma, seizures) induce loss of consciousness *via* the same mechanisms.

## Materials and Methods

The present report constitutes an entirely novel analysis of data that has been previously described with different methods [21]. Before detailing our analysis approach, based on graph-theoretic measures, we briefly describe the population, manipulation and data acquisition methods.

### Ethics statement

The study was approved by the Ethics Committee of the Medical School of the University of Liège (University Hospital, Liège, Belgium).

### Participants, conditions and fMRI data acquisition

#### Participants

The whole dataset included twenty healthy right-handed volunteers (16 female) between the ages of 18 and 31 (M = 22.40, SD = 3.40). However, given the strong sensitivity of graph-theoretic measures to motion [56], [57], subjects presenting any displacement above 3 mm throughout any one scan (of the 4 each subject underwent) was dropped. In total, 8 subjects were excluded due to movement, reducing our sample size to 12 volunteers. For the remaining volunteers average absolute movement was well below half a millimiter (0.33, 0.34, 0.20, 0.26, for W, S, LOC and R, respectively) and did not significantly differ across condition (1-way repeated measures ANOVA; 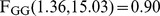; 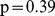). In addition, we remark that subject exclusion is, at present, a conservative strategy. Indeed, on the one hand, motion has been shown to affect graph theoretic measures [56], [57]. On the other hand, it has been shown that standard motion correction algorithms are insufficient corrections, and are liable to introduce spurious, but systematic, correlations [57]. Finally, novel more aggressive correction approaches censoring time-points [57], while correcting for motion and potential spurious correlations, are associated with the negative effects of reducing the number of time-points [65], [66] which has been shown to be associated with increases in the likelihood of high correlation, and to be a potential source of across condition or across subject bias [66]. As detailed below, in the following analysis we factor out motion as has been done previously [16], [25].

#### Conditions

Volunteers underwent four resting-state fMRI scans. The across-scan variable was the level of consciousness, as clinically evaluated by the Ramsay scale [67]. The first scan was accomplished with participants being fully awake (wakefulness; W). In the second scan participants were sedated (S; Ramsay level 3) so that while their response to verbal command were slowed, they were still present. In the third scan participants experienced loss of consciousness (LOC; Ramsay levels 5–6), and exhibited no response to verbal instruction. Finally, the last scan was performed after participants had recovered (R; Ramsay level 2).

#### fMRI data acquisition

Functional images were acquired on a 3 Tesla Siemens Allegra scanner (Siemens AG, Munich, Germany) with an Echo Planar Imaging sequence in 32 ascending slices (TR = 2,460 ms, TE = 40 ms, FOV = 220 mm, voxel size 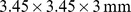, and matrix size 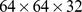). Because participants were acquired with a different number of volumes (varying between 196 and 350), correlation matrices were computed only on the first 196 volumes (8 min) for all conditions and subjects. For each participant one T1-weighted MP-RAGE image was also acquired (TR = 2,250 ms, TE = 2.99 ms, FOV = 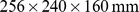, 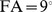, resolution 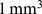 isovoxel).

### fMRI data analysis

Data analysis was carried out in three stages: initial preprocessing, support vector machine (SVM) matrix classification, and computation of global and local graph-theoretic measures.

#### Preprocessing

Functional and anatomical images were preprocessed according to the general procedures available in the 1000 Functional Connectome Project (http://fcon_1000.projects.nitrc.org/), and followed very closely the procedures employed in previous studies on the topic [16], [25]. First, the initial 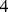 TRs of each functional dataset was removed. Second, data underwent slice-time correction, rigid-body adjustment for intra-run motion, brain extraction, 4 mm FWHM smoothing, band-pass filtering (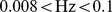), and removal of linear and quadratic trends. Nuisance signals, including motion parameters, white matter and CSF associated time-courses were partialled-out using a linear regression. Consistent with previous research [25], [37], global signal was not removed since it has been shown to be liable to introducing artifactual anti-correlations that can bias correlations differently in different parts of the brain depending on the underlying true interregional correlation structure, potentially introducing structure even where there is none [68], [69] and has been shown to suppress meaningful neural activity [70], [71], while regression of white matter and CSF signals has been shown to reduce many of the unwanted sources of noise that global signal regression is often used for [72]. Furthermore, with respect to graph theory analyses specifically, global signal regression has been reported to decrease reproducibility of both local and global topologically metrics [73]–[75]. The residuals of the regression were then co-registered to an MNI-space template (via 2-step registration using 6 degrees of freedom (dof) for within subject alignment of functional data to anatomical data and 12 dof to align the subject's anatomical image to the template). As part of this latter step, data were also resampled at 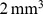 isovoxel resolution.

#### Brain network constructions

For each subject and each condition we constructed a graph representing a mathematical description of the brain as a functional network. A graph consists of a set of points and a set of lines connecting pairs of points [31]. In our framework, each point, also referred to as a *node* or *vertex*, corresponds to a specific brain region, or region of interest (ROI). Each line, also referred to as an *edge* or *link*, specifies the presence/absence of a connection between any two vertices and, for weighted graphs, the magnitude of the connection. (In our analysis the connection is the Pearson correlation 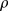 statistic between each pair of nodes.) In what follows graphs are typically visualized in a familiar matrix heat-map presentation, where each row/column of the square matrix represents an ROI, and element (*i*,*j*) specifies the functional correlation between 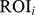 and 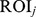.

**Figure 7:**
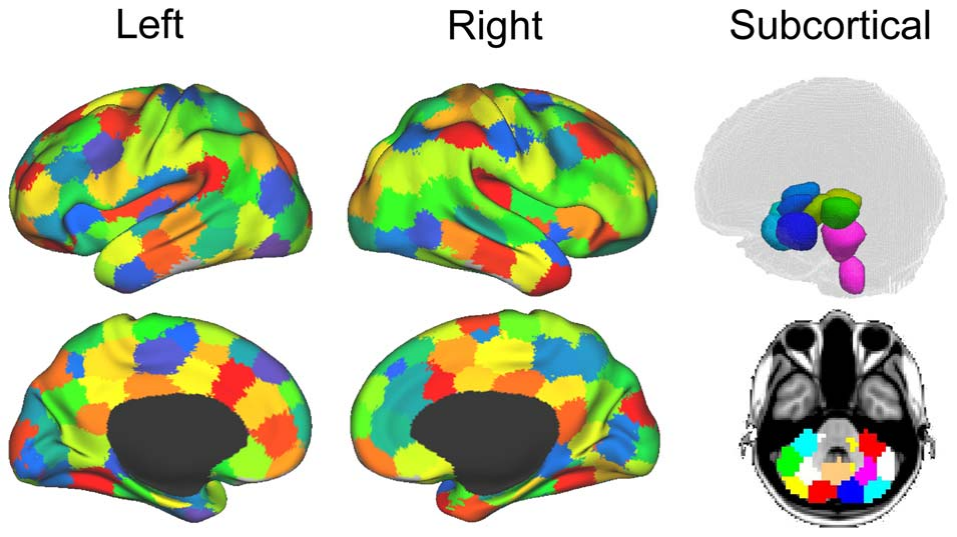
ROI selection. Parcellation of brain data into 194 cortical, subcortical and cerebellar ROIs.

Brain graphs were constructed in three steps. First, each individual data-set was parceled into 194 ROIs spanning cortex, sub-cortical nuclei, cerebellum and brainstem (see Figure 7). ROIs were defined independently, on the basis of a functional atlas that groups together spatially coherent voxels with homogeneous functional connectivity, at a desired resolution [76]. Specifically, we took the brain parcellation made available by Craddock and colleagues and employed that parcellation scheme to divide the brain in a set of 194 regions of interest. It is important to note that this procedure ensures that ROI selection is entirely independent of our data, thereby avoiding any form of bias in the analysis [77]. With the exception of atlas choice, this procedure follows exactly what has been done in previous research [25], [37], [78]. Choice of this parcellation scheme over previously used atlases such as the AAL [79] and the Harvard-Oxford Atlas [80], [81] is advantageous from several points of view. First, being functionally defined it clusters spatially proximal voxels by homogeneity of functional connections as opposed to clustering by anatomical position which, as exemplified by the case of the precentral gyrus ROIs in both the AAL and the Harvard-Oxford atlases, clusters together functionally very distinct subregions. Second, at our chosen level the Craddock ROIs have, collectively, almost twice the granularity as either structural atlas (i.e., 194 ROIs versus, 90 and 112 for the AAL and Harvard-Oxford atlases, respectively). Furthermore, when all atlases are equally resampled at 2 mm resolution, the ROIs obtained from the Craddock atlas appear much more homogeneous, in terms of size, than the other two (e.g., min to max voxel count range: 517, 5,005 and 8,076 for the Craddock, Harvard-Oxford and AAL atlases, respectively). After the functional parcellation of the brain into 194 ROIs, the average time-course associated with all voxels within each region was extracted. Finally, the average signal derived from each ROI was correlated to all other ROIs (using a Pearson's correlation coefficient; 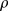) in order to obtain a 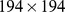 square connectivity matrix (i.e., the graph). These connectivity matrices were the input for the following two analyses.

#### Network classification

Classifications were performed, on a pairwise basis, using the Pattern Recognition of Brain Image Data software package (PROBID; http://www.kcl.ac.uk/iop/depts/neuroimaging/research/imaginganalysis/Software/PROBID.aspx). PROBID employs a linear kernel Support Vector Machine (SVM) [82] algorithm to achieve classification; that is, it attempts to find a hyperplane that separates the matrices according to their class membership (e.g., W vs. S). This approach is particularly appealing in the domain of brain imaging because of its well-known benign properties in circumstances in which the number of input features (here, the number of elements of the 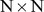 adjacency matrix) exceeds the number of samples [83]. Use of a linear SVM algorithm has two important advantages. First, it reduces the probability of over-fitting the data (as compared to non-linear algorithms, since the linear kernel has only one parameter that controls the trade-off between having zero training errors and allowing mis-classifications; cf. [83], [84]); second, it allows direct extraction of the weight vector that defines the hyperplane, that is, a vector specifying which elements are most relevant for discriminating between the groups. In our context, the input to the classifier were the unthresholded correlation matrices, implying that ROI-to-ROI correlations were the features upon which classifications were performed. Following the requirements of the software, each subject's adjacency matrix was vectorized; conversely, the classification results were reshaped into the original 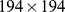 matrix form. Classification performance was evaluated using an exhaustive “leave-one-out cross validation” scheme, as implemented in PROBID, resulting in mean accuracy, mean sensitivity, and mean specificity percentages for each classification. In this approach, N different training datasets are built by leaving out, in each, a different subject. The estimated SVM hyperparameter (i.e., the hyperplane best separating the two classes) from each training set is then separately validated against the one dataset that has been left out of the training. Finally, the results from each separate iteration of the training-validation procedure are averaged. Overall, it is important to stress that our approach avoids the most frequent pitfalls known to potentially bias machine learning approaches (see [83] for a detailed discussion). First, because feature extraction (i.e., computation of the adjacency matrices) was performed on a single subject basis, our subject-based cross validation strategy adheres to the independence assumption of training and validation set, thereby ensuring that (i) the estimate of the hyperparameter is indeed inferred solely on the properties of the training set, and has not been contaminated by the test set, and that (ii) the model evaluation is not biased by any information included in the training set. Second, because our rejection of artifacts (e.g., modeling of motion) is unconnected to class membership, there is no risk that the rejection schema might bias the classification results. Similarly, the decision to entirely reject a subject whenever one of the four sessions presented excessive motion also allows us to avoid the potential biases associated with unbalanced class frequencies. Finally, the use of an exhaustive leave-one-out validation strategy provides an almost unbiased estimate of the generalization error [83], albeit at the expense of an increased variance of the estimator, thereby avoiding the possibility of excessively optimistic estimates of the generalization error. Overall, five classifications were computed. First, we attempted to classify the two states of wakefulness (W & R) versus the two states of altered consciousness (S & LOC). Then, we performed three pairwise classifications aimed at distinguishing contiguous states; namely, W vs. S; S vs. LOC; LOC vs. R. Finally, we compared the two states of wakefulness to each other (i.e., W vs. R). The statistical significance of each classification accuracy was assessed with a non-parametric approach based on repeated label permutation (10,000 times). The p-value from the permutation was derived by counting the number of times the true (that is, non-permuted) accuracy was greater than the accuracy derived after permuting the labels and dividing by 10,000. Familywise error rate was controlled for with a Bonferroni correction.

To visualize the results of the SVM classification for each comparison, we created “importance maps” (also referred to as “evidence maps”) [85], [86]. These maps, depicted in Figure 3, were obtained in two steps. First, we separated the elements of the weighting matrix *w*, which defines the hyperplane best separating two classes (e.g., LOC *versus* S), into two sparse matrices. The first sparse matrix, 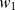, included all the elements contributing to classifying the first condition (e.g., LOC); the second sparse matrix,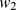, included all elements contributing to classifying the second condition (e.g., S). (It should be noted that while there is no absolute scale for quantitatively interpreting the importance maps, the greater the value for a given link, the greater its influence on the classification.) Second, we multiplied each sparse matrix by the average adjacency matrix across all subjects correctly classified. The resulting sparse matrices, which are depicted in Figure 3, thus show the average value, across all subjects correctly classified, of each edge contributing to the classification of a given condition scaled by how much it contributed. Positive values therefore indicate positive correlations (i.e., edges) contributing to a classification, scaled by how much they contributed to the classification; similarly, negative values indicate negative correlations (i.e., edges) contributing to a classification, scaled by how much they contributed to the classification.

In parallel to the SVM algorithm, all classifications were also computed using PROBID's Gaussian Process Classifier (GPC) [87] algorithm, which yielded nearly identical results.

Finally, to assess whether the pattern of connections which allowed classifying the transition from wakefulness to sedation (i.e., S vs. W) differed from the pattern of connections which allowed correctly classifying the transition from sedation to loss of consciousness (i.e., LOC vs. S), we performed a 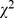 test comparing the distribution of classifying connections in the two comparisons. More in detail, for each of the two classifications, we divided the top 1% of edges contributing to correct classification into 15 kinds of connections (e.g., cortico-cortical (positive), cortico-cortical (negative), thalamo-cortical, striato-cortical, etc; see Tables S1 and S2). Counts for the two conditions were then entered into a contingency table and thus submitted to a 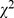 test comparing the two distributions.

#### Network analysis

To characterize local and global properties of the brain networks during normal wake, sedation, loss of consciousness and recovery, we employed a set of metrics derived from graph-theory [27], [54], [78]. Prior to the analysis, connectivity matrices were thresholded to avoid the possibility that weak and non-significant links representing spurious connections may obscure the topology of strong and significant connections [28]. Thresholding was performed on a proportional basis thus retaining, for each individual matrix, only the strongest *t*% (positive) edges. To date there is no agreed algorithm to select a unique threshold at which to perform graph-analysis, hence we followed one of the many procedures employed previously. Specifically, the lower boundary was selected to ensure the averaged degree was not smaller than 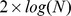, where N is the total number of nodes in the graph (i.e., N = 194). This lower boundary guaranteed that the resulting networks were estimable networks [30]. The upper boundary was selected to ensure that mean small-worldness (see below for the definition) in each condition was not smaller than 1.0. These two constraints yield an interval of density thresholds between 11% and 31%, which we sampled in steps of 5% (resulting in the following five density thresholds: 11%, 16%, 21%, 26%, 31%). The issue of thresholding is addressed in more detail in the Discussion.

Several global and local metrics were computed for each connectivity matrix, employing the Brain Connectivity Toolbox (BCT) [28]. First, we assessed large-scale network functional integration of brain networks, which captures the extent to which information from distributed regions can be rapidly combined, by measuring their characteristic path length and global efficiency. The *characteristic path length (L)* of a network is defined as the average length of the path uniting each pair of nodes within the network [30]. Since our networks are based on functional data, paths can be conceived as sequences of statistical associations (i.e., correlations) between nodes rather than physical existing/non-existing paths as might be the case with structural measures. The most common and mainstream interpretation of characteristic path length in networks is as a measure for global integration of information between topologically distant brain regions (see Discussion for more details). To date, the vast majority of graph analysis of functional data have been performed on binary graphs, that is, connectivity matrices in which all elements that survive thresholding are set to 1, regardless of their original value, and all other elements are set to 0. In this procedure, however, once matrices are binarized weak edges exert the same influence on network measures than strong edges. In the context of path length, for example, where strongly correlated nodes can be intuitively interpreted as (functionally) “closer,” binarization might result in erroneous creation of long-distance shortcuts, which can obscure the architecture of strong connections and, ultimately, important across-group differences [38]. Thus, following the development of metrics that make use of weight information [88], [89], we employ “weighted” measures (denoted, in the following formulae by the superscript ‘
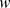
’). Characteristic path length was thus computed as follows:
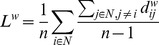
where 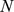 represents the set of all nodes within a network, 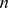 the number of nodes, and 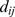 is the shortest path uniting nodes 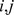. The average inverse of L is thus a measure of *global efficiency (* 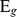 *)* of a network [39]. A different aspect of a network is the degree to which it exhibits functional segregation, which captures the extent to which subgroups of nodes densely interconnected with each other can carry out specialized processing. To quantify the presence of clusters of regions (e.g., modules) with strong statistical dependencies suggestive of segregated neural processing we computed the *clustering coefficient (C)*, a measure of the ‘cliquishness’ of networks' subdivisions defined as the average fraction of a node's neighbors that are also neighbors of each other [30]. In its weighted version, clustering is defined as [89]:
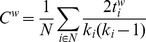
where 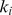 is the degree of a node (i.e., the sum of the weights of all nodes connecting to node 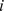), and 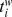 represents the geometric mean of all triangles (i.e., triads of reciprocally connected nodes) surrounding node 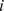. Similarly, *modularity (Q)* measures the extent to which a network can be optimally subdivided in a number of non-overlapping groups of regions which have high within-group connectivity and low across-group connectivity [90]. As for above, we employed the weighted version of the algorithm [88]:
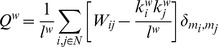
where 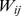 is the weight of the connection (i.e., correlation) between nodes 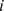 and 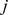, 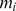 designates the module 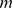 within which node 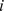 is contained, and 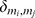 is equal to 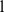 if nodes 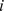 and 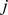 are part of the same module 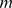, and 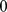 otherwise. To address the issue of degeneracy, we followed a three step procedure previously suggested [28]. Specifically, computation of modularity was refined by first applying a finetuning algorithm to the above calculation, and then running a final probabilistic tuning algorithm on the finetuned result. In addition, because the calculation of modularity is heuristic and variable across iterations, we performed the three steps above (modularity, fine-tuning and probabilistic-tuning) 50 times for each matrix, and then employed the mean modularity value across the 50 iterations for subsequent analyses (we note that we also performed the analyses on the median modularity, across the 50 iterations, and it yield qualitatively identical results).

It is important to note that it is often not possible to interpret the significance of the absolute values of the above metrics. Thus, the metrics derived for each network are typically normalized by the average metric derived from 100 random networks (with matched size and degree distribution). The normalized characteristic path length (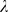) is thus the ratio of the characteristic path length (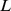) of a brain network and the average characteristic path length of 100 random networks (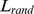). Normalized clustering coefficient (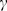), global efficiency (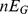) and modularity (nQ) are defined analogously. In addition, the ratio of normalized clustering and normalized characteristic path length is often referred to as the small world property (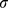) of a network. Small-world networks are networks that are neither completely random nor completely regular; rather, they lie in-between these two extremes and exhibit a high degree of clustering, like regular networks, and a low characteristic path length, like random networks [30]. Several studies to date have observed small-world characteristics in the human brain [26], [91].

In addition to the metrics above, we also characterized the brain networks on a node-wise basis. First, we computed the *strength (* 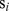 *)* of each node, a basic measure of node centrality defined as:
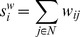
Nodes with a high strength are nodes that are (functionally) close to many other nodes in the graph. In general, measures of centrality are based on the intuition that nodes participating in several short paths within a network are important controls of information flow [31]. Node strength can therefore be interpreted as an index of communication activity within a network. Finally, we also calculated the *local efficiency (* 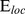 *)* of each node to get a local measure of segregation. In its weighted version, local efficiency for node *i* is defined as [28], [39]:
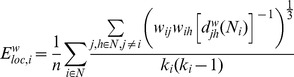
where 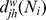 is the length of the shortest (weighted) path between 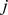 and 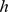 that contains only neighbors of 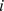. This metric can be interpreted as measuring how “fault tolerant” the system is since it captures how efficient the communication is between the first neighbors of 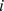 when 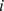 is removed [39]. The present extension of the local efficiency to weighted networks differentiates the influence on presumed fault tolerance of strongly connected and weakly connected neighbors, and broadly parallels a generalization of the clustering coefficient to weighted networks [28], [89].

Statistical analyses were performed in Stata (SE, Version 13, Stata Corp) and Matlab (Mathworks Matlab, version R2013a). The effect of condition on each metric was assessed using a repeated measures ANOVA model with condition (4 levels: W, S, LOC and R) and threshold (5 levels: 11%, 16%, 21%, 26%, 31%) as factors. Following convention [92], where the Machuly's test indicated violation of the sphericity assumption, degrees of freedom were calculated with the Huynh-Feldt correction for moderate violations (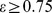; denoted with the 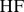 subscript) and the Greenhouse-Geisser for more severe violations (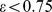; denoted with the 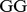 subscript). Because of the relatively small size of our sample, we also report the partial omega-squared (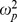), a measure of effect size in the population that is resistant to sample size and therefore provides a better measure of the magnitude of the effect between conditions [93], [94]. Unlike other metrics of effect size that are significantly upwards biased (e.g., 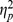), 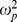 does not results in inflated estimates even for small sample sizes [93], [95]. This statistic is usually interpreted as the percent of the dependent variable's variance accounted for by the effect in the population with other non-error sources of variance being partialled out, and is considered to indicate, for values of 0.01, 0.06, 0.14, small, medium and large effects, respectively [96]. Pairwise comparisons among the different conditions were performed on the basis of estimated means using a Sidak adjustment for multiple comparisons. The 2-way repeated measures ANOVA was followed-up with a set of 1-way repeated measures ANOVA, one per each threshold, with condition as the only factor. For global measures, significance was assessed against a conventional 0.05 p-value criterion. For local measures, due to the multiple comparisons issue, we employed an FDR-adjusted criterion of 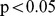 (following [97]).

## Supporting Information

Figure S1
**Classification results for S vs. R and W vs. LOC.** Top 1% connections contributing to the group SVM classification of (A) S vs. R, and (B) W vs. LOC. For each comparison, the upper triangle shows the connections contributing to correctly classifying the first condition, the lower triangle shows the connections contributing to correctly classifying the second condition. Red connections indicate positive correlations contributing to the correct classification of a condition, blue connections indicate negative correlations contributing to the correct classification of a condition. Classification of S vs. R achieved 71% accuracy (58% sensitivity, and 83% specificity; p = 0.01), while classification of W vs. LOC achieved 87% accuracy (83% sensitivity, and 92% specificity; 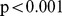). Abbreviations: 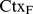: cortex, frontal lobe; 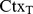: cortex, temporal lobe; 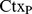: cortex, parietal lobe; 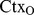: cortex, occipital lobe; BG: basal ganglia; Thl: thalamus; BS: brainstem; Crbl: cerebellum.(TIF)

Table S1 **SVM classification (I): Top 1% of classifying connections.** Count (percent positive, percent negative) of the number of edges contributing to correctly classifying all states of wakefulness (i.e., W&R) as compared to all states of sedation (i.e., S&LOC). (See Figure 3a, main text.)
(PDF)

Table S2 **SVM classification (II): Top 1% of classifying connections.** Count (percent positive, percent negative) of the number of edges contributing to correctly classifying S vs. W, LOC vs. S, and R vs. LOC. (See Figure 3bcd and Figure 4, main text.)
(PDF)
