# Supplementary figures and images for: Dynamic Change of Global and Local Information Processing in Propofol-Induced Loss and Recovery of Consciousness
Source: PLoS Comput Biol. 2013 Oct 17;9(10):e1003271. doi: 10.1371/journal.pcbi.1003271 (PMC3798283; doi:10.1371/journal.pcbi.1003271)

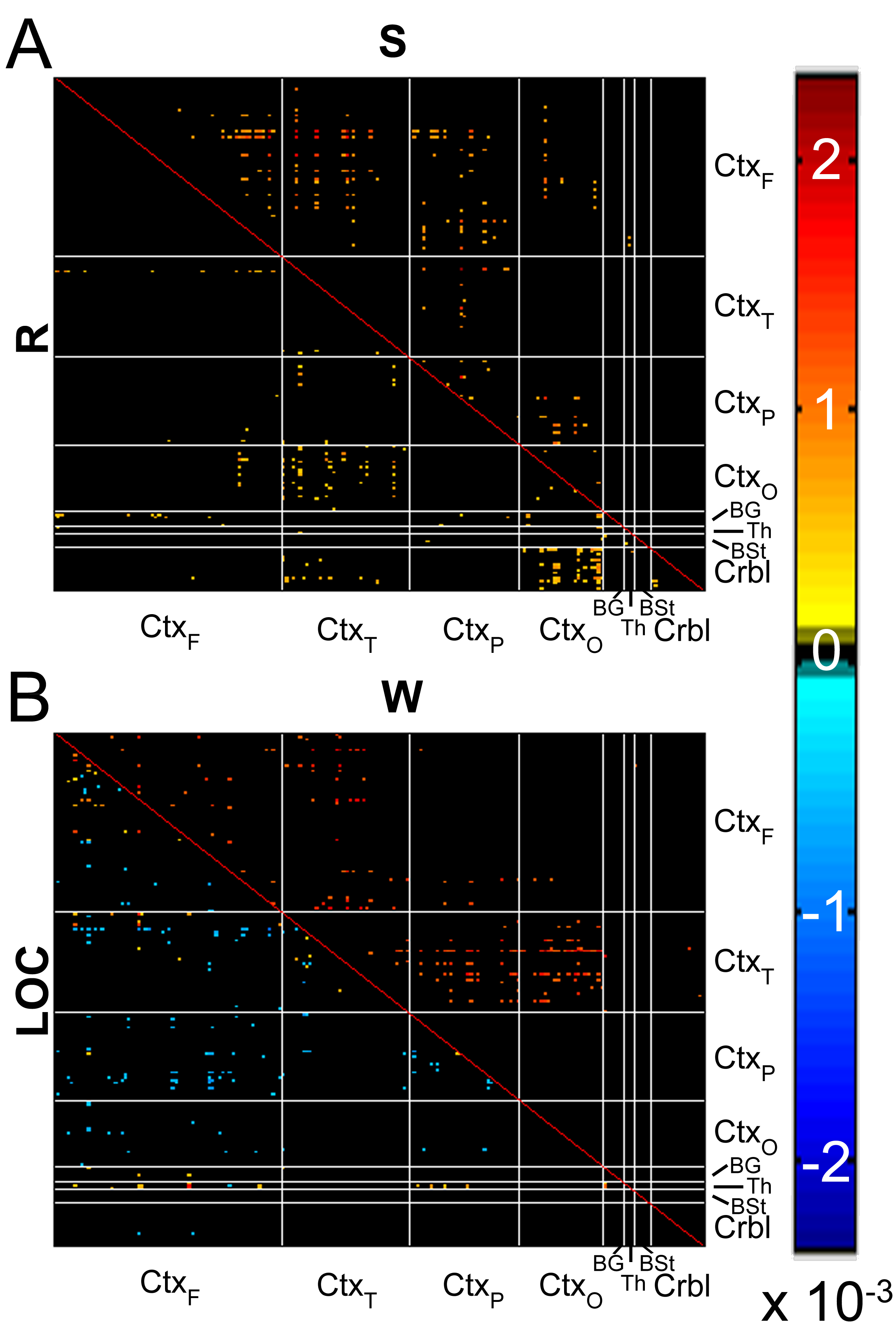

Supplement: Figure S1 — Classification results for S vs. R and W vs. LOC. Top 1% connections contributing to the group SVM classification of (A) S vs. R, and (B) W vs. LOC. For each comparison, the upper triangle shows the connections contributing to correctly classifying the first condition, the lower triangle shows the connections contributing to correctly classifying the second condition. Red connections indicate positive correlations contributing to the correct classification of a condition, blue connections indicate negative correlations contributing to the correct classification of a condition. Classification of S vs. R achieved 71% accuracy (58% sensitivity, and 83% specificity; p = 0.01), while classification of W vs. LOC achieved 87% accuracy (83% sensitivity, and 92% specificity; ). Abbreviations: : cortex, frontal lobe; : cortex, temporal lobe; : cortex, parietal lobe; : cortex, occipital lobe; BG: basal ganglia; Thl: thalamus; BS: brainstem; Crbl: cerebellum. (TIF) [file pcbi.1003271.s001.tif]
