# Supplementary material for: Dynamic Change of Global and Local Information Processing in Propofol-Induced Loss and Recovery of Consciousness
Source: PLoS Comput Biol. 2013 Oct 17;9(10):e1003271. doi: 10.1371/journal.pcbi.1003271 (PMC3798283; doi:10.1371/journal.pcbi.1003271)
Supplement: Table S1 — SVM classification (I): Top 1% of classifying connections. Count (percent positive, percent negative) of the number of edges contributing to correctly classifying all states of wakefulness (i.e., W&R) as compared to all states of sedation (i.e., S&LOC). (See Figure 3a, main text.) (PDF) [file pcbi.1003271.s002.pdf]

	**(W&R) vs. (S&LOC)** Count (%pos, %neg)	**(S&LOC) vs. (W&R)** Count (%pos, %neg)
Cortico-Cortical	178 (100, 0)	137 (59, 41)
Fronto-Frontal	1 (100, 0)	25 (96, 4)
Fronto-Temporal	27 (100, 0)	29 (66, 34)
Fronto-Parietal	1 (100, 0)	26 (58, 42)
Fronto-Occipital	11 (100, 0)	8 (13, 88)
Temporo-Temporal	12 (100, 0)	10 (60, 40)
Temporo-Parietal	29 (100, 0)	11 (64, 36)
Temporo-Occipital	96 (100, 0)	1 (0, 100)
Parieto-Parietal		9 (56, 44)
Parieto-Occipital		10 (30, 70)
Occipito-Occipital	1 (100, 0)	8 (13, 88)
Striato-Cortical	1 (100, 0)	4 (100, 0)
Striato-Frontal	1 (100, 0)	1 (100, 0)
Striato-Temporal		
Striato-Parietal		2 (100, 0)
Striato-Occipital		1 (100, 0)
Thalamo-Cortical	1 (100, 0)	40 (95, 5)
Thalamo-Frontal		15 (87, 13)
Thalamo-Temporal		6 (100, 0)
Thalamo-Parietal		18 (100, 0)
Thalamo-Occipital	1 (100, 0)	1 (100, 0)
Tronco-Cortical		1 (100, 0)
Tronco-Frontal		
Tronco-Temporal		1 (100, 0)
Tronco-Parietal		
Tronco-Occipital		
Cerebello-Cortical	6 (100, 0)	3 (0, 100)
Cerebello-Frontal		2 (0, 100)
Cerebello-Temporal	4 (100, 0)	1 (0, 100)
Cerebello-Parietal		
Cerebello-Occipital	2 (100, 0)	
Striato-Striatal		
Thalamo-Striatal		2 (100, 0)
Tronco-Striatal		
Cerebello-Striatal		
Thalamo-Thalamic		
Tronco-Thalamic		
Cerebello-Thalamic	1 (100, 0)	
Tronco-TroncoEncephalic		
Cerebello-TroncoEncephalic		
Cerebello-Cerebellar		
