# Supplementary material for: Dynamic Change of Global and Local Information Processing in Propofol-Induced Loss and Recovery of Consciousness
Source: PLoS Comput Biol. 2013 Oct 17;9(10):e1003271. doi: 10.1371/journal.pcbi.1003271 (PMC3798283; doi:10.1371/journal.pcbi.1003271)
Supplement: Table S2 — SVM classification (II): Top 1% of classifying connections. Count (percent positive, percent negative) of the number of edges contributing to correctly classifying S vs. W, LOC vs. S, and R vs. LOC. (See Figure 3bcd and Figure 4, main text.) (PDF) [file pcbi.1003271.s003.pdf]

	**S vs. W** Count (%pos, %neg)	**LOC vs. S** Count (%pos, %neg)	**R vs. LOC** Count (%pos, %neg)
Cortico-Cortical	102 (100, 0)	158 (11, 89)	182 (100, 0)
Fronto-Frontal	48 (100, 0)	19 (42, 58)	30 (100, 0)
Fronto-Temporal	27 (100, 0)	28 (21, 79)	16 (100, 0)
Fronto-Parietal	16 (100, 0)	24 (0, 100)	18 (100, 0)
Fronto-Occipital	1 (100, 0)	25 (0, 100)	3 (100, 0)
Temporo-Temporal	2 (100, 0)	6 (50, 50)	7 (100, 0)
Temporo-Parietal	1 (100, 0)	1 (0, 100)	30 (100, 0)
Temporo-Occipital		2 (0, 100)	74 (100, 0)
Parieto-Parietal	4 (100, 0)	15 (0, 100)	2 (100, 0)
Parieto-Occipital	2 (100, 0)	36 (0, 100)	2 (100, 0)
Occipito-Occipital	1 (100, 0)	2 (0, 100)	
Striato-Cortical	1 (100, 0)	16 (100, 0)	2 (100, 0)
Striato-Frontal	1 (100, 0)	8 (100, 0)	1 (100, 0)
Striato-Temporal		3 (100, 0)	1 (100, 0)
Striato-Parietal		1 (100, 0)	
Striato-Occipital		4 (100, 0)	
Thalamo-Cortical	76 (100, 0)	6 (100, 0)	
Thalamo-Frontal	37 (100, 0)	6 (100, 0)	
Thalamo-Temporal	10 (100, 0)		
Thalamo-Parietal	29 (100, 0)		
Thalamo-Occipital			
Tronco-Cortical	7 (100, 0)		
Tronco-Frontal	3 (100, 0)		
Tronco-Temporal	1 (100, 0)		
Tronco-Parietal	3 (100, 0)		
Tronco-Occipital			
Cerebello-Cortical	1 (100, 0)	5 (100, 0)	3 (100, 0)
Cerebello-Frontal		3 (100, 0)	
Cerebello-Temporal	1 (100, 0)	1 (100, 0)	3 (100, 0)
Cerebello-Parietal			
Cerebello-Occipital		1 (100, 0)	
Striato-Striatal			
Thalamo-Striatal		2 (100, 0)	
Tronco-Striatal			
Cerebello-Striatal			
Thalamo-Thalamic			
Tronco-Thalamic			
Cerebello-Thalamic			
Tronco-TroncoEncephalic			
Cerebello-TroncoEncephalic			
Cerebello-Cerebellar			
